# A Retrospective Study of Genetic Characterization in Suspected Visceral Leishmaniasis Cases in Greece, 2005 to 2020

**DOI:** 10.3390/pathogens13080688

**Published:** 2024-08-14

**Authors:** Maria Evangelidou, Sofia Makka, Ioanna Papadogiannaki, Myrto Koutantou, Nikolaos Tegos, Anastasia Mpimpa, Eleni Patsoula, Emmanouil Angelakis

**Affiliations:** 1 Diagnostic Department and Public Health Laboratories, Hellenic Pasteur Institute, 11521 Athens, Greece; meuagelidou@gmail.com (M.E.); makkasofia@gmail.com (S.M.); ioannapap97@gmail.com (I.P.); myrto.koutantou@gmail.com (M.K.); 2 Unit of Parasitic and Tropical Infections, Laboratory for the Surveillance of Infectious Diseases (LSID), Division of Infectious, Parasitic Diseases and Zoonoses, Department of Public Health Policy, School of Public Health, University of West Attica, 11521 Athens, Greece; ntegos@uniwa.gr (N.T.); nmpimpa@uniwa.gr (A.M.); epatsoula@uniwa.gr (E.P.)

**Keywords:** genotyping, Greece, ITS type A, *Leishmania infantum*, seasonality, immunocompromised

## Abstract

*Leishmania infantum* is considered the predominant *Leishmania* species responsible for visceral leishmaniasis (VL) in Greece but limited molecular-typing-based studies have been performed so far. We retrospectively analyzed data and serum samples collected from 3661 individuals suspected for VL in a sixteen-year period, from 2005 to 2020, to study the seasonality and demographic characteristics of VL cases and to define the *L. infantum* genotypes circulating in the country. Serum samples were tested with immunofluorescence assay and/or molecular assay. qPCR *Leishmania*-positive samples were subjected to genotypic analysis based on polymorphisms in 12 microsatellite regions of the internal transcribed spacers (ITSs) 1 and 2. We diagnosed 219 definite (6%, sample with a positive molecular assay and/or antibody titer ≥ 1:400) and 230 probable (6.3%, sample with antibody titer between 1:100 and 1:200) VL cases. Data analysis revealed that amongst VL-definite cases, the age group (≥65) constitutes the most affected factor, since 36.9% of the VL cases belonged to this age group. Amongst the VL definite cases, the most frequently reported symptoms were fever (83%), splenomegaly (49%), and hepatomegaly (40%), but this was not the case for immunocompromised patients that developed non-typical symptoms of leishmaniasis. Although no statistically significant differences in the overall seasonality of VL cases were observed, February and June showed a significantly higher proportion of VL cases compared to August and December. Genotyping of ITS1 and ITS2 regions revealed that all VL cases belong to ITS type A of *L. infantum.* Our study provides epidemiological information on VL and demonstrates for the first time, providing genotypic data, the circulation of ITS type A *L. infantum* in Greece.

## 1. Introduction

Leishmaniasis is a parasitic, vector-borne disease, caused by intracellular protozoan parasites belonging to the genus *Leishmania* [1]. It may be zoonotic or anthroponotic, and transmission occurs through the bite of an infected female phlebotomine sand fly [2]. Clinical features of the disease include a broad range of symptoms with different degrees of severity that depend upon the involved *Leishmania* species and the host immune response [3]. Manifestations range from the localized cutaneous (CL) to the visceral (VL) form, which causes a systematic disease with potentially fatal outcomes if left untreated [3]. According to the World Health Organization (WHO), leishmaniasis is one of the seven most important tropical diseases, posing a serious problem to global health [3]. Approximately 0.9 to 1.6 million new cases and 20,000 to 30,000 deaths are recorded, while more than 12 million people are infected and 350 million are at risk of acquiring the disease worldwide [4], annually. Leishmaniasis is present in all continents except Oceania, and it has become endemic in North Africa, Asia, the Middle East, Central and South America, and in the Mediterranean region [3]. This global spread has been related to factors concerning environmental, demographic, and human behavioral changes [2].

In the Mediterranean region, four *Leishmania* species have been found to cause visceral and/or cutaneous disease (*L. infantum*, *L. tropica*, *L. major*, and *L. donovani*) [2]. Specifically, in Greece, *L. infantum* is considered the predominant species responsible for VL [5]. Although most data on leishmaniasis in Greece are provided by case reports, two studies revealed that human VL does not display any seasonal characteristics and cases were uniformly distributed throughout the year [6,7]. To the best of our knowledge, there are limited data from molecular-typing-based studies concerning *Leishmania* in our country; therefore, our objective is to identify the circulation of *L. infantum* genotypes in Greece and study the seasonality of human VL using data collected during a sixteen-year period.

## 2. Materials and Methods

### 2.1. Study Design and Patients

The results obtained from serum samples of patients with suspected VL were retrospectively analyzed. Serum samples that were included in the present study arrived at the National Reference Laboratory for Leishmaniasis in Hellenic Pasteur Institute, from January 2005 to December 2020 (*n* = 3661). Fifty-two (52) *Leishmania*-positive DNA samples extracted from serum samples from patients diagnosed from June 2018 to June 2019 at the specialized Unit of Parasitic and Tropical Diseases, University of West Attica (UNIWA) were processed for genotypic and phylogenetic analysis. The Hellenic Pasteur Institute routinely receives specimens from hospitalized patients and outpatients with suspected zoonotic infections from all over Greece, contributing effectively to disease diagnosis and epidemiology. A total of 3661 individuals with suspected leishmaniasis were analyzed, for 3565 of which an acute-phase sample was obtained, and for the remaining 96 individuals, both acute- and convalescent-phase samples (collected >2 weeks after the onset of symptoms) were obtained. For all of the serum samples mentioned above, immunofluorescence assay and/or molecular assay were performed for the diagnosis of leishmaniasis. Furthermore, demographic information (age and gender) and clinical symptoms of leishmaniasis were recorded and analyzed and subsequent seasonal analysis of the leishmaniasis cases was performed.

### 2.2. Case Definition

A suspected leishmaniasis case is considered positive for VL when a positive molecular assay and/or antibody titer ≥ 1:400 of a single serum is obtained. A suspected leishmaniasis case is considered probable when a single serum antibody titer is between 1:100 and 1:200 and it is negative for the presence of *Leishmania* DNA. A suspected leishmaniasis case is considered negative when a single serum antibody titer is <1:100 and it is negative for the presence of *Leishmania* DNA.

### 2.3. L. infantum Cells Preparation for Immunofluorescence Assay (IFA)

*L. infantum* (MON-1, MCAN/PT/98/IMT 244) antigen was produced as previously described with slight modifications [8]. In brief, promastigotes of *L. infantum* were centrifuged at 1300× *g* for 10 min at 4 °C; the resulting pellet was rinsed three times in phosphate-buffered saline (PBS, pH 7.2–7.4) and then centrifuged at 350× *g* for 15 min at room temperature. The final cell pellet was resuspended in PBS to adjust it to 3–4 × 10^6^ promastigotes/mL. For immunofluorescence assay (IFA), slides were coated with 10 μL of antigen at room temperature overnight [8].

### 2.4. Immunofluorescence Assay (IFA)

Patients’ sera were tested for the presence of *Leishmania*-specific antibodies against *L. infantum* by IFA. In brief, the slides were incubated with 10 μL serial two-fold dilutions (1:25 to 1:3200) of the serum samples at 37 °C for 30 min, washed in PBS for 10 min, and air dried. The slides were then incubated with 10 μL of diluted fluorescein isothiocyanate (FITC)-conjugated anti-human IgG at 37 °C for 30 min, washed, mounted with buffered glycerin, and processed for fluorescence microscopy [8]. The highest dilution showing a positive signal for fluorescent promastigotes is the antibody titer of the serum sample.

### 2.5. Molecular Assay

DNA was extracted from the serum samples using a QIAamp DNA Mini Kit (Qiagen, Hilden, Germany), according to the manufacturers’ instructions, under sterile conditions to avoid cross-contamination. All DNA samples extracted from serum samples were then tested by quantitative real-time PCR (qPCR) [9,10], targeting kinetoplast DNA for the detection of *L. infantum* [11]. Quality control of extracted DNA was performed in all DNA samples tested [12].

### 2.6. Cohen’s Kappa Coefficient

The qualitative agreement between the two diagnostic techniques (IFA and qPCR) was evaluated using Cohen’s kappa coefficient, which is a robust statistic metric [13]. It ranges between 0 and 1; values ≤ 0 indicate no agreement, 0.01–0.20 indicates none to slight, 0.21–0.40 indicates fair, 0.41–0.60 indicates moderate, and 0.61–0.80 indicates substantial, while a value between 0.81 and 1.00 signifies almost perfect agreement [14].

### 2.7. Genotyping and Phylogenetic Analysis

Serum samples that were found to be positive for *Leishmania* DNA were further subjected to sequencing analysis by targeting two different regions of the ribosomal DNA, internal transcribed spacers (ITSs) 1 and 2. ITS1 (320 bp) and ITS2 (740 bp) regions were amplified and sequenced using the primer pairs LITSR/L5.8S and L5.8SR/LITSV, respectively, as previously described [15]. The sequences were concatenated and a specific ITS type (A–H) was assigned based on polymorphisms of twelve microsatellite regions (four regions in ITS1 and eight regions in ITS2) [15]. Phylogenetic tree analysis was performed using the maximum likelihood method with 1000 bootstrap replications in MEGA 11.0 software [16]. Phylogenetic trees were constructed using representative nucleotide sequences of the eight distinct ITS types (A–H), as previously described for the *L. donovani* complex [15], which were retrieved from GenBank database.

### 2.8. Statistical Analysis 

The chi-squared test was applied for calculating statistically significant differences both in the number and proportions of VL cases. Comparisons were made across different groups of VL cases distributed among months, seasons, and age groups. Seasons were defined as winter (January–March), spring (April–June), summer (July–September), and autumn (October–December). Statistical analysis on the age of VL patients was conducted across the following age groups: ≤22, 23–45, 46–64, and ≥65 years old. A *p* value < 0.05 was considered statistically significant. Data were analyzed using GraphPad Prism 9 software. 

### 2.9. Ethics Approval Statement

This study is based on routine diagnosis samples. All collected data were anonymized in standardized forms according to the Ethic and Scientific Committee of the Hellenic Pasteur Institute under registration numbers EIP-GDPR-E01.01 and 4806/31-07-2024. 

## 3. Results

### 3.1. VL Cases 

We analyzed 3661 individuals with suspected leishmaniasis, for 3565 of which an acute-phase sample was obtained, and for the remaining 96 individuals, both acute- and convalescent-phase samples were obtained. From the 3565 acute-phase serum samples, 3339 were tested only with IFA, 146 only with qPCR, and 80 samples were tested with both methods. From ninety-six convalescence serum samples, ninety-three were tested only with IFA, two only with qPCR, and one sample with both methods. A total of two hundred nineteen (6%) definite positive leishmaniasis cases were diagnosed, from which two hundred sixteen leishmaniasis cases were diagnosed at the acute phase of the disease symptoms and three leishmaniasis cases at the convalescence phase. From the 219 definite positive leishmaniasis cases, 163 (74.4%) cases were diagnosed with IFA for the presence of *Leishmania* antibodies (not tested with qPCR), 33 (15.1%) cases with qPCR for the presence of *Leishmania* DNA (not tested with IFA), and 23 (10.5%) cases with both assays (Table 1). 

From both acute and convalescence groups, 81 serum samples were tested with both assays and 23 patients were diagnosed positive for leishmaniasis; among them, nineteen patients were found positive in both assays, one patient was IFA-positive and qPCR-negative, three patients were IFA-negative and PCR-positive, and fifty-eight were negative in both techniques (Table 2). A comparison of these diagnostic techniques in terms of level of agreement using Cohen’s kappa coefficient revealed that the degree of agreement was κ = 0.87, indicating an almost perfect agreement between them.

Furthermore, data on sex were available for 204 (204/219, 93.8%) of the VL cases and data analysis revealed that the majority of them were males (53.9%), without any significant difference from females (46.1%) (*p* > 0.05). Analysis on age as a risk factor for leishmaniasis showed that the mean (SD) age of patients was 53.7 (±26.7) years, while the median age was 57 years. Among the 149 (149/219, 68%) VL cases for which age information was known, 55 (55/149, 36.9%) belonged to the age group ≥65 years old, followed by the age groups 46–64 years old (42/149, 28.2%), ≤22 years old (28/149, 18.8%), and 23–45 years old (24/149, 16.1%). Statistical analysis revealed that the VL cases that belong to the age group ≥65 years old were significantly higher compared to those belonging to the age group 23–45 years old (*p* = 0.037) (Table 3). Lastly, 230 (230/3661, 6.3%) suspected cases for leishmaniasis were assigned as probable cases.

### 3.2. Clinical Manifestations

Clinical information was available for 35 patients (35/219, 16%), and for 14 (14/35, 40%) of them hospitalization was necessary. The most frequently reported symptom was fever (83%), followed by splenomegaly (49%) and hepatomegaly (40%). Other less common manifestations include weight loss (23%), anemia (23%), leukopenia (20%), and thrombocytopenia (20%). Seven patients (7/35, 20%) were immunocompromised either due to kidney transplantation (*n* = 4), cancer (*n* = 2), or HIV infection (*n* = 1), and interestingly, four of them belonged to the groups that reported absence of fever (6/35, 17%) and hepatosplenomegaly (21/35, 60%).

### 3.3. Seasonality

**Figure 1:**
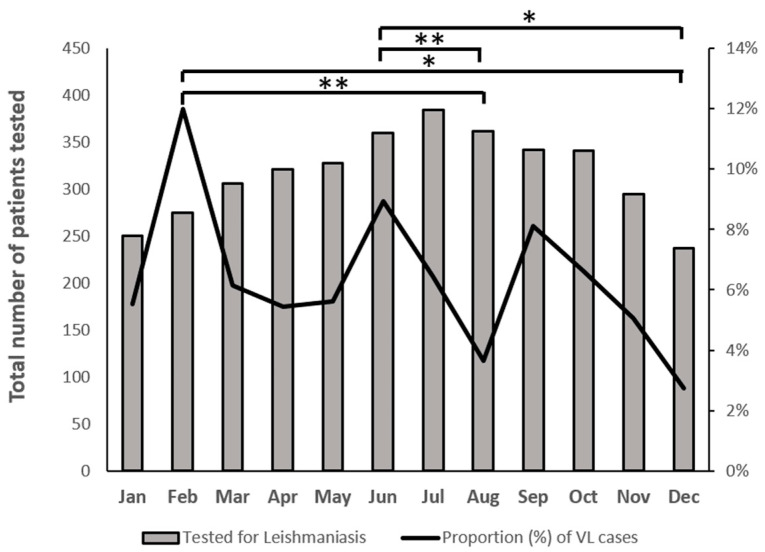
Total number of patients tested and distribution of proportion of VL cases across months for the sixteen-year period 2005–2020. * *p* < 0.05; ** *p* < 0.005.

The proportions of VL cases and the total number of patients tested during the sixteen-year period were plotted for each month to identify seasonal variations (Figure 1). The median number (±SD) of positive incidents by month was eighteen (±5.6), ranging from eight to twenty-nine. Although the proportion of cases did not differ significantly among seasons and no clear seasonal pattern was observed, peaks were observed in February (12%) and June (9%). August and December had the lowest proportion of positive VL cases (3.6% and 2.7%, respectively). Statistical analysis revealed that the VL cases that occurred in February were significantly higher compared to the ones that occurred in August and December (*p* = 0.002 and *p* = 0.013, respectively). The same also applies for the VL cases that occurred in June, which were found to be significantly higher compared to the ones that occurred in August and December (*p* = 0.005 and *p* = 0.02, respectively). No statistically significant differences were observed among the total number of patients tested for each month. 

### 3.4. Phylogenetic Tree

**Figure 2:**
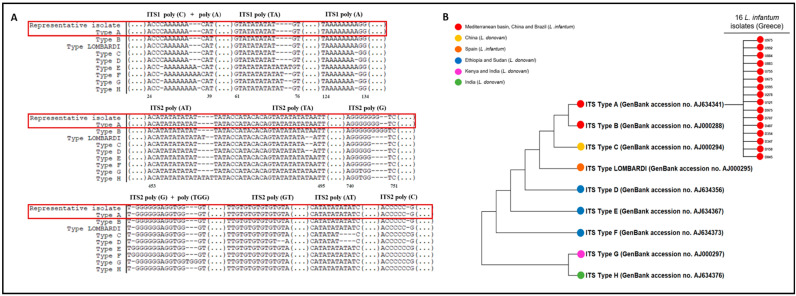
Sequencing and phylogenetic analysis of *L infantum* based on ITS regions. (**A**) Alignment of ITS reference types and one representative of 16 Greek isolates. Red boxes indicate absolute similarity of one representative Greek isolate with ITS Type A. (**B**) Phylogenetic relationships of *L. donovani* complex strains and 16 Greek isolates. The phylogenetic tree was constructed by using the maximum-likelihood method based on the Tamura–Nei substitution model. Colored bullets show the species and corresponding geographical regions of ITS type origins.

A total of 75 *L. infantum* serum samples (23/75 from the National Reference Laboratory for Leishmaniasis in Hellenic Pasteur Institute and 52/75 from the specialized Unit of Parasitic and Tropical Diseases, University of West Attica) with a positive qPCR were further processed for genotyping analysis to identify their ITS type and 27 of them (27/75, 36%) had sufficient DNA loads. ITS sequencing analysis was successful in 16 of them (16/27, 59%). Finally, the phylogenetic analysis based on the 12 variable sites of the concatenated sequences classified all strains into ITS type A (Figure 2A,B). The concatenated and aligned sequences of the 16 samples that were successfully analyzed, together with the LOMBARDI type and the eight ITS-based types (A–H) with their GenBank Accession No., are available in the Supplementary Material (Document S1).

## 4. Discussion

We retrospectively analyzed data collected from 3661 individuals suspected for VL in a sixteen-year period, from 2005 to 2020, to study the seasonality and demographic characteristics and to provide epidemiological data on VL cases in Greece. Furthermore, we genetically analyzed qPCR leishmania-positive serum samples from the same individuals to define the *L. infantum* genotypes circulating in the country. Serology is the most commonly used method for the diagnosis of leishmaniasis, but in certain cases where antibody titer falls between 1:100 and 1:200, it is difficult for clinicians to reach a definite diagnosis. To eliminate this possibility, we have been using molecular assays since 2013, thus the number of serum samples tested with qPCR is lower compared to the ones tested with IFA. Nevertheless, using Cohen’s kappa to measure inter-rater reliability, we indicated almost perfect agreement between the two diagnostic techniques.

Although most data on human leishmaniasis in Greece are provided as case reports, a few seroepidemiological and surveillance studies have been performed in our country. Specifically, since 1985, seroepidemiology studies in Greece have been performed in several regions of the country, with seropositivity rates ranging from 0.5% in Epirus (1994–2001) [17] to 15% in Lasithi (2003) [18]. According to the National Mandatory Notification System (National Public Health Organization-NPHO), the mean annual incidence rates of reported VL cases between 1998 and 2011 and between 2004 and 2018 were 0.34 and 0.49 per 100.000 population, respectively [6,7]. The increased positivity rate among suspected VL cases reported in the present study, and by others [5], can be explained by the fact that more sensitive molecular assays have been widely introduced in routine diagnosis. In addition, physicians are becoming more and more familiar with the clinical manifestations of the disease compared to the previous years. Data analysis on sex revealed that the majority of VL cases were males (53.9%), without any significant difference from females (46.1%), a finding that has also been previously reported in two other surveillance studies [6,7]. Our data analysis on age revealed that the age group ≥65 years old is the most affected one (36.9%), which is in agreement with another Greek study [19], whereas the literature reports that young age can serve as a risk factor for leishmaniasis [20]. In our study, the age group ≤22 years old accounts for the 18.8% of VL cases, and a possible reason for this could be the smaller number of patients tested compared to other age groups (Table 1).

Similar studies report that the clinical manifestations of VL may vary from asymptomatic to full-blown disease accompanied by fever, hepatosplenomegaly, and cytopenias. In our patient cohort, very few patients (35/219, 16%) participating in this study reported symptoms; nevertheless, the symptoms mentioned above were the most frequently symptoms reported in the present study, confirming similar percentages in clinical manifestations reported in previous studies [19]. Since one major risk factor is immunosuppression, seven patients (7/35, 20%) were immunocompromised either due to kidney transplantation, cancer, or HIV infection and four of them reported an absence of typical symptoms like fever and hepatosplenomegaly. Considering that normal body temperature is rarely reported in immunocompetent patients with VL, our study reinforces the previously reported finding in HIV patients that immunocompromised patients may not develop typical clinical manifestations of leishmaniasis [21], and this is something that both clinicians and laboratory scientists must bear in mind.

As mentioned, Greece is an endemic country for VL, with *L. infantum* being the predominant species [2,5,6,19]. Furthermore, disease incident rates and seasonal distribution have also been recorded in several neighboring and other European countries, including Albania, Bulgaria, Montenegro, Malta, and Spain. To date, surveillance studies conducted in Greece and Malta for the periods 1981–2018 and 2004–2008, respectively, have revealed no clear seasonal patterns in VL cases in either country [6,7,22]. Indeed, overall seasonal fluctuations have not been observed in our study either, although February and August were characterized by a statistically higher proportion of VL cases. However, epidemiological data from the above-mentioned countries are contradictory, with Albania and Montenegro [23,24] showing that most cases appeared predominantly in spring–summer, whereas in Bulgaria and Spain, cases were observed more frequently during the cold months of the year [25,26]. Although sand flies are active during warm months, the increase in global temperature due to climate change highly affects larval survival in winter, expanding the seasonal dynamics of the vector. In addition, late January to early February is a period of intense sunshine and increased temperature in our country (Alkyonides days), which could explain the significantly increased proportion of VL cases observed in February in the present study.

In the past few years, different typing methods have been described in epidemiological and population studies of the *L. donovani* complex, including PCR-restriction fragment length polymorphism (PCR-RFLP), multilocus enzyme electrophoresis (MLEE), multilocus microsatellite typing (MLST), multilocus sequence typing (MLMT) analysis, and sequencing of specific genes (ITS1, kDNA, the mini-exon, hsp70, and k26) [27,28]. Among them, MLMT seems to have higher discriminatory power, giving new insights into the circulation of strains. In the current study, we performed a microsatellite-based analysis using ITS1 and ITS2 regions, as have been previously employed by other laboratories [15,29]. Analysis of the polymorphic sites revealed the presence of ITS type A in all samples successfully sequenced. As there is no previous evidence of circulating types of *Leishmania* in Greece, we compared our results with similar studies conducted in the Mediterranean basin. According to MLMT data, ITS type A had originally been observed in *L. infantum* strains isolated in France, Italy, and Spain, while a few years later, this specific genotype was detected, along with ITS-LOMBARDI, in the southwest of Madrid, Spain during an outbreak of the disease (2009–2012) that caused more than 400 reported cases [15,29].

## 5. Conclusions

In conclusion, we provide, for the first time, evidence that ITS type A is the only circulating genotype of *L. infantum* in Greece and that there is no seasonal trend in VL cases throughout the seasons, although February and June are characterized by a statistically significantly higher proportion of VL cases. Furthermore, clinicians should be aware of non-typical manifestations of the disease, especially in immunocompromised patients. As VL poses a serious threat to public health, especially in endemic countries like Greece, we emphasize the importance of an accurate and constant molecular typing surveillance system, which will offer a better understanding of the epidemiology of the disease and potentially prevent the spreading of new *Leishmania* species from other parts of the world.

## Figures and Tables

**Table 1:** Distribution of total serum samples tested in the acute and convalescence phase of the disease and corresponding number of VL cases for each phase. The number of total serum samples tested with IFA, qPCR, and both techniques and the corresponding number of VL cases diagnosed with each technique are given.

Clinical Phase	Total No. of Tested	Total No. of Positives	Types of Assays					
			IFA		qPCR		IFA and qPCR	
			No. of Tested	No. of Positives	No. of Tested	No. of Positives	No. of Tested	No. of Positives
Acute	3565	216	3339	160	146	33	80	23
Convalescence	96	3	93	3	2	0	1	0
Total	3661	219	3432	163	148	33	81	23

**Table 2:** Level of agreement between IFA and qPCR using Cohen’s kappa statistic.

	IFA	Negative	Positive	Total
qPCR				
Negative		58	1	59
Positive		3	19	22
Total		61	20	81
Kappa coefficient		κ = 0.87		

**Table 3:** Distribution and analysis of total patients and corresponding VL cases by demographic criteria of sex and age. Age groups with statistically significant difference have an asterisk (*).

	Sex			Age Groups				
	Male	Female	Unknown	≤22	23–45	46–64	≥65	Unknown
Total No. of tested	1568	1706	387	296	604	626	827	1308
Total No. of positives	110	94	15	28	24	42	55	70
Proportion of positives in total no tested (%)	7	5.5	3.9	9.5	4	6.7	6.7	5.4
No. of cases with available information on sex and age	204			149				
Proportion of positives in cases with available information (%)	53.9	46.1		18.8	16.1 *	28.2	36.9 *	
*p* value	*p* > 0.05			* *p* = 0.037				

* Statistically significant difference was observed in the indicated age groups.

## Data Availability

All reference ITS-based type sequences used in the present study can be found at GenBank. The original contributions presented in the study are included in the Supplementary Material; further inquiries can be directed to the corresponding author.

## Supplementary Materials

The following supporting information can be downloaded at: https://www.mdpi.com/article/10.3390/pathogens13080688/s1, Document S1: Concatenated and aligned ITS1 and ITS2 sequences used in the present study.
