# Supplementary material for: A Retrospective Study of Genetic Characterization in Suspected Visceral Leishmaniasis Cases in Greece, 2005 to 2020
Source: Pathogens. 2024 Aug 14;13(8):688. doi: 10.3390/pathogens13080688 (PMC11357202; doi:10.3390/pathogens13080688)
Supplement: Supplementary file 1 [file pathogens-13-00688-s001.zip › pathogens-3098921-supplementary.pdf]

Supplementary Data S1

Concatenated and aligned ITS1 and ITS2 sequences used in the present study

>ITS Type A (GenBank accession no. AJ634341)

CTGGATCATTTTCCGATGATTACACCCAAAAAA---
ATATACAACTCGGGGAGACCTATGTATATATAT-----
GTAGGCCTTTCCCACATACACAGCAAAGTTTTGTACTCAAAATTTGCAGTA
AAAAAAAGGCCGAT
CGACGTTATAACGCACCGCCTATACAAAAGCAAAAATGTCCGTTTATACA
AAAAATATACGGCGTTTCGGTTTTTGGCGGGGTGGGTGCGTGTGTGGATA
ACGGCTCACATAACGTGTCGCGATGGATGACTTGGCTTCCTATTTCGTTGA
AGAACGCAGTAAAGTGCGATAAGTGGTATCAATTGCAGAATCATTCAATT
ACCGAATCTTTGAACGCAAACGGCGCATGGGAGAAGCTCTATTGTGTCAT
CCCCGTGCATGCCATATTCTCAGTGTCGAACAAAAAACAACACGCCGCCT
CCTCTCTTCTGCACATATATATAT---TATACCATACA
CAGTATATATATAATTATGTGTTGGAAGCCAAGAGGAGGCGTGTGTTTGT
GTTGTGCGCATATTATATGTATATATGCTGTGTGCACACGTAGACAAGTTA
GAGTTGGACAAATACACACATGCACTCTCTTTTGTGTGGGTGCGCGCGTG
GAAACTCCTCTCTGGTGCTTGCAAAGCAGTCTTTTTCTCTTTCTCTTTTTCT
CTCTCCATTCTCTCCTCTCTTTTTTCATCAAAAAGGGGGGAGAGAAAAAGA
GAGAGGAGGGGGGG--
TCGAGGGAGAGAGGCTGTGACCAGGATTATTAAACAAAAAACCAAAACG
AGAATTCAACTTCGCGTTGGCCATTTTTTGCTTAAT-GGGGGGAGGTGG---
GTGTGGGTGGTGTGTGGCTCTCTCTCTGTGTGGT
ATATATATATGTATATTAGAAGTAGGTTGTGTGTGTGTGTATGTGTTTTAC
ACATATATATATCCGCGCCCTCACTCTCTCATATATAATTTATATGTACGC
ACAGAGAAAAAAGAGAGGCGCTCTCTTTTCCCCCCACCCCC-
GACAACCTTTGTTTACAGACCTGAGTGT

>D045

CTGGATCATTTTCCGATGATTACACCCAAAAAA---
ATATACAACTCGGGGAGACCTATGTATATATAT-----
GTAGGCCTTTCCCACATACACAGCAAAGTTTTGTACTCAAAATTTGCAGTA
AAAAAAAGGCCGAT
CGACGTTATAACGCACCGCCTATACAAAAGCAAAAATGTCCGTTTATACA
AAAAATATACGGCGTTTCGGTTTTTGGCGGGGTGGGTGCGTGTGTGGATA
ACGGCTCACATAACGTGTCGCGATGGATGACTTGGCTTCCTATTTCGTTGA
AGAACGCAGTAAAGTGCGATAAGTGGTATCAATTGCAGAATCATTCAATT
ACCGAATCTTTGAACGCAAACGGCGCATGGGAGAAGCTCTATTGTGTCAT
CCCCGTGCATGCCATATTCTCAGTGTCGAACAAAAAACAACACGCCGCCT
CCTCTCTTCTGCACATATATATAT---TATACCATACA
CAGTATATATATAATTATGTGTTGGAAGCCAAGAGGAGGCGTGTGTTTGT
GTTGTGCGCATATTATATGTATATATGCTGTGTGCACACGTAGACAAGTTA
GAGTTGGACAAATACACACATGCACTCTCTTTTGTGTGGGTGCGCGCGTG

GAAACTCCTCTCTGGTGCTTGCAAAGCAGTCTTTTTCTCTTTCTCTTTTTCT
CTCTCCATTCTCTCCTCTCTTTTTTCATCAAAAAGGGGGGAGAGAAAAAGA
GAGAGGAGGGGGGG--
TCGAGGGAGAGAGGCTGTGACCAGGATTATTAAACAAAAAACCAAAACG
AGAATTCAACTTCGCGTTGGCCATTTTTTGCTTAAT-GGGGGGAGGTGG---
GTGTGGGTGGTGTGTGGCTCTCTCTCTGTGTGGT
ATATATATATGTATATTAGAAGTAGGTTGTGTGTGTGTGTATGTGTTTTAC
ACATATATATATCCGCGCCCTCACTCTCTCATATATAATTTATATGTACGC
ACAGAGAAAAAAGAGAGGCGCTCTCTTTTCCCCCCACCCCC-
GACAACCTTTGTTTACAGACCTGAGTGT

>D158

CTGGATCATTTTCCGATGATTACACCCAAAAAA---
ATATACAACTCGGGGAGACCTATGTATATATAT-----
GTAGGCCTTTCCCACATACACAGCAAAGTTTTGTACTCAAAATTTGCAGTA
AAAAAAAGGCCGAT
CGACGTTATAACGCACCGCCTATACAAAAGCAAAAATGTCCGTTTATACA
AAAAATATACGGCGTTTCGGTTTTTGGCGGGGTGGGTGCGTGTGTGGATA
ACGGCTCACATAACGTGTCGCGATGGATGACTTGGCTTCCTATTTCGTTGA
AGAACGCAGTAAAGTGCGATAAGTGGTATCAATTGCAGAATCATTCAATT
ACCGAATCTTTGAACGCAAACGGCGCATGGGAGAAGCTCTATTGTGTCAT
CCCCGTGCATGCCATATTCTCAGTGTCGAACAAAAAACAACACGCCGCCT
CCTCTCTTCTGCACATATATATAT---TATACCATACA
CAGTATATATATATAATTATGTGTTGGAAGCCAAGAGGAGGCGTGTGTTTGT
GTTGTGCGCATATTATATGTATATATGCTGTGTGCACACGTAGACAAGTTA
GAGTTGGACAAATACACACATGCACTCTCTTTTGTGTGGGTGCGCGCGTG
GAAACTCCTCTCTGGTGCTTGCAAAGCAGTCTTTTTCTCTTTCTCTTTTTCT
CTCTCCATTCTCTCCTCTCTTTTTTCATCAAAAAGGGGGGAGAGAAAAAGA
GAGAGGAGGGGGGG--
TCGAGGGAGAGAGGCTGTGACCAGGATTATTAAACAAAAAACCAAAACG
AGAATTCAACTTCGCGTTGGCCATTTTTTGCTTAAT-GGGGGGAGGTGG---
GTGTGGGTGGTGTGTGGCTCTCTCTCTGTGTGGT
ATATATATATGTATATTAGAAGTAGGTTGTGTGTGTGTGTATGTGTTTTAC
ACATATATATATCCGCGCCCTCACTCTCTCATATATAATTTATATGTACGC
ACAGAGAAAAAAGAGAGGCGCTCTCTTTTCCCCCCACCCCC-
GACAACCTTTGTTTACAGACCTGAGTGT

>D347

CTGGATCATTTTCCGATGATTACACCCAAAAAA---
ATATACAACTCGGGGAGACCTATGTATATATAT-----
GTAGGCCTTTCCCACATACACAGCAAAGTTTTGTACTCAAAATTTGCAGTA
AAAAAAAGGCCGAT
CGACGTTATAACGCACCGCCTATACAAAAGCAAAAATGTCCGTTTATACA
AAAAATATACGGCGTTTCGGTTTTTGGCGGGGTGGGTGCGTGTGTGGATA
ACGGCTCACATAACGTGTCGCGATGGATGACTTGGCTTCCTATTTCGTTGA
AGAACGCAGTAAAGTGCGATAAGTGGTATCAATTGCAGAATCATTCAATT
ACCGAATCTTTGAACGCAAACGGCGCATGGGAGAAGCTCTATTGTGTCAT

CCCCGTGCATGCCATATTCTCAGTGTCGAACAAAAAACAACACGCCGCCT
CCTCTCTTCTGCACATATATATAT---TATACCATACA
CAGTATATATATAATTATGTGTTGGAAGCCAAGAGGAGGCGTGTGTTTGT
GTTGTGCGCATATTATATGTATATATGCTGTGTGCACACGTAGACAAGTTA
GAGTTGGACAAATACACACATGCACTCTCTTTTGTGTGGGTGCGCGCGTG
GAAACTCCTCTCTGGTGCTTGCAAAGCAGTCTTTTTCTCTTTCTCTTTTTCT
CTCTCCATTCTCTCCTCTCTTTTTTCATCAAAAAGGGGGGAGAGAAAAAGA
GAGAGGAGGGGGGG--
TCGAGGGAGAGAGGCTGTGACCAGGATTATTAAACAAAAAACCAAAACG
AGAATTCAACTTCGCGTTGGCCATTTTTTGCTTAAT-GGGGGGAGGTGG---
GTGTGGGTGGTGTGTGGCTCTCTCTCTGTGTGGT
ATATATATATGTATATTAGAAGTAGGTTGTGTGTGTGTGTATGTGTTTTAC
ACATATATATATCCGCGCCCTCACTCTCTCATATATAATTTATATGTACGC
ACAGAGAAAAAAGAGAGGCGCTCTCTTTTCCCCCCACCCCC-
GACAACCTTTGTTTACAGACCTGAGTGT

>D354

CTGGATCATTTTCCGATGATTACACCCAAAAAA---
ATATACAACTCGGGGAGACCTATGTATATATAT-----
GTAGGCCTTTCCCACATACACAGCAAAGTTTTGTACTCAAAATTTGCAGTA
AAAAAAAGGCCGAT
CGACGTTATAACGCACCGCCTATACAAAAGCAAAAATGTCCGTTTATACA
AAAAATATACGGCGTTTCGGTTTTTGGCGGGGTGGGTGCGTGTGTGGATA
ACGGCTCACATAACGTGTCGCGATGGATGACTTGGCTTCCTATTTCGTTGA
AGAACGCAGTAAAGTGCGATAAGTGGTATCAATTGCAGAATCATTCAATT
ACCGAATCTTTGAACGCAAACGGCGCATGGGAGAAGCTCTATTGTGTCAT
CCCCGTGCATGCCATATTCTCAGTGTCGAACAAAAAACAACACGCCGCCT
CCTCTCTTCTGCACATATATATAT---TATACCATACA
CAGTATATATATAATTATGTGTTGGAAGCCAAGAGGAGGCGTGTGTTTGT
GTTGTGCGCATATTATATGTATATATGCTGTGTGCACACGTAGACAAGTTA
GAGTTGGACAAATACACACATGCACTCTCTTTTGTGTGGGTGCGCGCGTG
GAAACTCCTCTCTGGTGCTTGCAAAGCAGTCTTTTTCTCTTTCTCTTTTTCT
CTCTCCATTCTCTCCTCTCTTTTTTCATCAAAAAGGGGGGAGAGAAAAAGA
GAGAGGAGGGGGGG--
TCGAGGGAGAGAGGCTGTGACCAGGATTATTAAACAAAAAACCAAAACG
AGAATTCAACTTCGCGTTGGCCATTTTTTGCTTAAT-GGGGGGAGGTGG---
GTGTGGGTGGTGTGTGGCTCTCTCTCTGTGTGGT
ATATATATATGTATATTAGAAGTAGGTTGTGTGTGTGTGTATGTGTTTTAC
ACATATATATATCCGCGCCCTCACTCTCTCATATATAATTTATATGTACGC
ACAGAGAAAAAAGAGAGGCGCTCTCTTTTCCCCCCACCCCC-
GACAACCTTTGTTTACAGACCTGAGTGT
>D487

CTGGATCATTTTCCGATGATTACACCCAAAAAA---
ATATACAACTCGGGGAGACCTATGTATATATAT-----
GTAGGCCTTTCCCACATACACAGCAAAGTTTTGTACTCAAAATTTGCAGTA
AAAAAAAGGCCGAT

CGACGTTATAACGCACCGCCTATACAAAAGCAAAAATGTCCGTTTATACA
AAAAATATACGGCGTTTCGGTTTTTGGCGGGGTGGGTGCGTGTGTGGATA
ACGGCTCACATAACGTGTCGCGATGGATGACTTGGCTTCCTATTTCGTTGA
AGAACGCAGTAAAGTGCGATAAGTGGTATCAATTGCAGAATCATTCAATT
ACCGAATCTTTGAACGCAAACGGCGCATGGGAGAAGCTCTATTGTGTCAT
CCCCGTGCATGCCATATTCTCAGTGTCGAACAAAAAACAACACGCCGCCT
CCTCTCTTCTGCACATATATATAT---TATACCATACA
CAGTATATATATAATTATGTGTTGGAAGCCAAGAGGAGGCGTGTGTTTGT
GTTGTGCGCATATTATATGTATATATGCTGTGTGCACACGTAGACAAGTTA
GAGTTGGACAAATACACACATGCACTCTCTTTTGTGTGGGTGCGCGCGTG
GAAACTCCTCTCTGGTGCTTGCAAAGCAGTCTTTTTCTCTTTCTCTTTTTCT
CTCTCCATTCTCTCCTCTCTTTTTTCATCAAAAAGGGGGGAGAGAAAAAGA
GAGAGGAGGGGGGG--
TCGAGGGAGAGAGGCTGTGACCAGGATTATTAAACAAAAAACCAAAACG
AGAATTCAACTTCGCGTTGGCCATTTTTTGCTTAAT-GGGGGGAGGTGG---
GTGTGGGTGGTGTGTGGCTCTCTCTCTGTGTGGT
ATATATATATGTATATTAGAAGTAGGTTGTGTGTGTGTGTATGTGTTTTAC
ACATATATATATCCGCGCCCTCACTCTCTCATATATAATTTATATGTACGC
ACAGAGAAAAAAGAGAGGCGCTCTCTTTTCCCCCCACCCCC-
GACAACCTTTGTTTACAGACCTGAGTGT

>D707

CTGGATCATTTTCCGATGATTACACCCAAAAAA---
ATATACAACTCGGGGAGACCTATGTATATATAT-----
GTAGGCCTTTCCCACATACACAGCAAAGTTTTGTACTCAAAATTTGCAGTA
AAAAAAAGGCCGAT
CGACGTTATAACGCACCGCCTATACAAAAGCAAAAATGTCCGTTTATACA
AAAAATATACGGCGTTTCGGTTTTTGGCGGGGTGGGTGCGTGTGTGGATA
ACGGCTCACATAACGTGTCGCGATGGATGACTTGGCTTCCTATTTCGTTGA
AGAACGCAGTAAAGTGCGATAAGTGGTATCAATTGCAGAATCATTCAATT
ACCGAATCTTTGAACGCAAACGGCGCATGGGAGAAGCTCTATTGTGTCAT
CCCCGTGCATGCCATATTCTCAGTGTCGAACAAAAAACAACACGCCGCCT
CCTCTCTTCTGCACATATATATAT---TATACCATACA
CAGTATATATATAATTATGTGTTGGAAGCCAAGAGGAGGCGTGTGTTTGT
GTTGTGCGCATATTATATGTATATATGCTGTGTGCACACGTAGACAAGTTA
GAGTTGGACAAATACACACATGCACTCTCTTTTGTGTGGGTGCGCGCGTG
GAAACTCCTCTCTGGTGCTTGCAAAGCAGTCTTTTTCTCTTTCTCTTTTTCT
CTCTCCATTCTCTCCTCTCTTTTTTCATCAAAAAGGGGGGAGAGAAAAAGA
GAGAGGAGGGGGGG--
TCGAGGGAGAGAGGCTGTGACCAGGATTATTAAACAAAAAACCAAAACG
AGAATTCAACTTCGCGTTGGCCATTTTTTGCTTAAT-GGGGGGAGGTGG---
GTGTGGGTGGTGTGTGGCTCTCTCTCTGTGTGGT
ATATATATATGTATATTAGAAGTAGGTTGTGTGTGTGTGTATGTGTTTTAC
ACATATATATATCCGCGCCCTCACTCTCTCATATATAATTTATATGTACGC
ACAGAGAAAAAAGAGAGGCGCTCTCTTTTCCCCCCACCCCC-
GACAACCTTTGTTTACAGACCTGAGTGT

>D975

CTGGATCATTTCCGATGATTACACCCAAAAAA---
ATATACAACTCGGGGAGACCTATGTATATATAT-----
GTAGGCCTTTCCCACATACACAGCAAAGTTTTGTACTCAAAATTTGCAGTA
AAAAAAAGGCCGAT
CGACGTTATAACGCACCGCCTATACAAAAGCAAAAATGTCCGTTTATACA
AAAAATATACGGCGTTTCGGTTTTTGGCGGGGTGGGTGCGTGTGTGGATA
ACGGCTCACATAACGTGTCGCGATGGATGACTTGGCTTCCTATTTCGTTGA
AGAACGCAGTAAAGTGCGATAAGTGGTATCAATTGCAGAATCATTCAATT
ACCGAATCTTTGAACGCAAACGGCGCATGGGAGAAGCTCTATTGTGTCAT
CCCCGTGCATGCCATATTCTCAGTGTCGAACAAAAAACAACACGCCGCCT
CCTCTCTTCTGCACATATATATAT---TATACCATACA
CAGTATATATATAATTATGTGTTGGAAGCCAAGAGGAGGCGTGTGTTTGT
GTTGTGCGCATATTATATGTATATATGCTGTGTGCACACGTAGACAAGTTA
GAGTTGGACAAATACACACATGCACTCTCTTTTGTGTGGGTGCGCGCGTG
GAAACTCCTCTCTGGTGCTTGCAAAGCAGTCTTTTTCTCTTTCTCTTTTTCT
CTCTCCATTCTCTCCTCTCTTTTTTCATCAAAAAGGGGGGAGAGAAAAAGA
GAGAGGAGGGGGGG--
TCGAGGGAGAGAGGCTGTGACCAGGATTATTAAACAAAAAACCAAAACG
AGAATTCAACTTCGCGTTGGCCATTTTTTGCTTAAT-GGGGGGAGGTGG---
GTGTGGGTGGTGTGTGGCTCTCTCTCTGTGTGGT
ATATATATATGTATATTAGAAGTAGGTTGTGTGTGTGTGTATGTGTTTTAC
ACATATATATATCCGCGCCCTCACTCTCTCATATATAATTTATATGTACGC
ACAGAGAAAAAAGAGAGGCGCTCTCTTTTCCCCCCACCCCC-
GACAACCTTTGTTTACAGACCTGAGTGT

>U121

CTGGATCATTTCCGATGATTACACCCAAAAAA---
ATATACAACTCGGGGAGACCTATGTATATATAT-----
GTAGGCCTTTCCCACATACACAGCAAAGTTTTGTACTCAAAATTTGCAGTA
AAAAAAAGGCCGAT
CGACGTTATAACGCACCGCCTATACAAAAGCAAAAATGTCCGTTTATACA
AAAAATATACGGCGTTTCGGTTTTTGGCGGGGTGGGTGCGTGTGTGGATA
ACGGCTCACATAACGTGTCGCGATGGATGACTTGGCTTCCTATTTCGTTGA
AGAACGCAGTAAAGTGCGATAAGTGGTATCAATTGCAGAATCATTCAATT
ACCGAATCTTTGAACGCAAACGGCGCATGGGAGAAGCTCTATTGTGTCAT
CCCCGTGCATGCCATATTCTCAGTGTCGAACAAAAAACAACACGCCGCCT
CCTCTCTTCTGCACATATATATAT---TATACCATACA
CAGTATATATATAATTATGTGTTGGAAGCCAAGAGGAGGCGTGTGTTTGT
GTTGTGCGCATATTATATGTATATATGCTGTGTGCACACGTAGACAAGTTA
GAGTTGGACAAATACACACATGCACTCTCTTTTGTGTGGGTGCGCGCGTG
GAAACTCCTCTCTGGTGCTTGCAAAGCAGTCTTTTTCTCTTTCTCTTTTTCT
CTCTCCATTCTCTCCTCTCTTTTTTCATCAAAAAGGGGGGAGAGAAAAAGA
GAGAGGAGGGGGGG--
TCGAGGGAGAGAGGCTGTGACCAGGATTATTAAACAAAAAACCAAAACG
AGAATTCAACTTCGCGTTGGCCATTTTTTGCTTAAT-GGGGGGAGGTGG---
GTGTGGGTGGTGTGTGGCTCTCTCTCTGTGTGGT

ATATATATATGTATATTAGAAGTAGGTTGTGTGTGTGTGTATGTGTTTTAC
ACATATATATATCCGCGCCCTCACTCTCTCATATATAATTTATATGTACGC
ACAGAGAAAAAAGAGAGGCGCTCTCTTTTCCCCCCACCCCC-
GACAACCTTTGTTTACAGACCTGAGTGT

>U278

CTGGATCATTTTCCGATGATTACACCCAAAAAA---
ATATACAACTCGGGGAGACCTATGTATATATAT-----
GTAGGCCTTTCCCACATACACAGCAAAGTTTTGTACTCAAAATTTGCAGTA
AAAAAAAGGCCGAT
CGACGTTATAACGCACCGCCTATACAAAAGCAAAAATGTCCGTTTATACA
AAAAATATACGGCGTTTCGGTTTTTGGCGGGGTGGGTGCGTGTGTGGATA
ACGGCTCACATAACGTGTCGCGATGGATGACTTGGCTTCCTATTTCGTTGA
AGAACGCAGTAAAGTGCGATAAGTGGTATCAATTGCAGAATCATTCAATT
ACCGAATCTTTGAACGCAAACGGCGCATGGGAGAAGCTCTATTGTGTCAT
CCCCGTGCATGCCATATTCTCAGTGTCGAACAAAAAACAACACGCCGCCT
CCTCTCTTCTGCACATATATATAT---TATACCATACA
CAGTATATATATAATTATGTGTTGGAAGCCAAGAGGAGGCGTGTGTTTGT
GTTGTGCGCATATTATATGTATATATGCTGTGTGCACACGTAGACAAGTTA
GAGTTGGACAAATACACACATGCACTCTCTTTTGTGTGGGTGCGCGCGTG
GAAACTCCTCTCTGGTGCTTGCAAAGCAGTCTTTTTCTCTTTCTCTTTTTCT
CTCTCCATTCTCTCCTCTCTTTTTTCATCAAAAAGGGGGGAGAGAAAAAGA
GAGAGGAGGGGGGG--
TCGAGGGAGAGAGGCTGTGACCAGGATTATTAAACAAAAAACCAAAACG
AGAATTCAACTTCGCGTTGGCCATTTTTTGCTTAAT-GGGGGGAGGTGG---
GTGTGGGTGGTGTGTGGCTCTCTCTCTGTGTGGT
ATATATATATGTATATTAGAAGTAGGTTGTGTGTGTGTGTATGTGTTTTAC
ACATATATATATCCGCGCCCTCACTCTCTCATATATAATTTATATGTACGC
ACAGAGAAAAAAGAGAGGCGCTCTCTTTTCCCCCCACCCCC-
GACAACCTTTGTTTACAGACCTGAGTGT

>U595

CTGGATCATTTTCCGATGATTACACCCAAAAAA---
ATATACAACTCGGGGAGACCTATGTATATATAT-----
GTAGGCCTTTCCCACATACACAGCAAAGTTTTGTACTCAAAATTTGCAGTA
AAAAAAAGGCCGAT
CGACGTTATAACGCACCGCCTATACAAAAGCAAAAATGTCCGTTTATACA
AAAAATATACGGCGTTTCGGTTTTTGGCGGGGTGGGTGCGTGTGTGGATA
ACGGCTCACATAACGTGTCGCGATGGATGACTTGGCTTCCTATTTCGTTGA
AGAACGCAGTAAAGTGCGATAAGTGGTATCAATTGCAGAATCATTCAATT
ACCGAATCTTTGAACGCAAACGGCGCATGGGAGAAGCTCTATTGTGTCAT
CCCCGTGCATGCCATATTCTCAGTGTCGAACAAAAAACAACACGCCGCCT
CCTCTCTTCTGCACATATATATAT---TATACCATACA

CAGTATATATATAATTATGTGTTGGAAGCCAAGAGGAGGCGTGTGTTTGT
GTTGTGCGCATATTATATGTATATATGCTGTGTGCACACGTAGACAAGTTA
GAGTTGGACAAATACACACATGCACTCTCTTTTGTGTGGGTGCGCGCGTG
GAAACTCCTCTCTGGTGCTTGCAAAGCAGTCTTTTTCTCTTTCTCTTTTTCT
CTCTCCATTCTCTCCTCTCTTTTTTCATCAAAAAGGGGGGAGAGAAAAAGA
GAGAGGAGGGGGGG--
TCGAGGGAGAGAGGCTGTGACCAGGATTATTAAACAAAAAACCAAAACG
AGAATTCAACTTCGCGTTGGCCATTTTTTGCTTAAT-GGGGGGAGGTGG---
GTGTGGGTGGTGTGTGGCTCTCTCTCTGTGTGGT
ATATATATATGTATATTAGAAGTAGGTTGTGTGTGTGTGTATGTGTTTTAC
ACATATATATATCCGCGCCCTCACTCTCTCATATATAATTTATATGTACGC
ACAGAGAAAAAAGAGAGGCGCTCTCTTTTCCCCCCACCCCC-
GACAACCTTTGTTTACAGACCTGAGTGT

>U675

CTGGATCATTTTCCGATGATTACACCCAAAAAA---
ATATACAACTCGGGGAGACCTATGTATATATAT-----
GTAGGCCTTTCCCACATACACAGCAAAGTTTTGTACTCAAAATTTGCAGTA
AAAAAAAGGCCGAT
CGACGTTATAACGCACCGCCTATACAAAAGCAAAAATGTCCGTTTATACA
AAAAATATACGGCGTTTCGGTTTTTGGCGGGGTGGGTGCGTGTGTGGATA
ACGGCTCACATAACGTGTCGCGATGGATGACTTGGCTTCCTATTTCGTTGA
AGAACGCAGTAAAGTGCGATAAGTGGTATCAATTGCAGAATCATTCAATT
ACCGAATCTTTGAACGCAAACGGCGCATGGGAGAAGCTCTATTGTGTCAT
CCCCGTGCATGCCATATTCTCAGTGTCGAACAAAAAACAACACGCCGCCT
CCTCTCTTCTGCACATATATATAT---TATACCATACA
CAGTATATATATAATTATGTGTTGGAAGCCAAGAGGAGGCGTGTGTTTGT
GTTGTGCGCATATTATATGTATATATGCTGTGTGCACACGTAGACAAGTTA
GAGTTGGACAAATACACACATGCACTCTCTTTTGTGTGGGTGCGCGCGTG
GAAACTCCTCTCTGGTGCTTGCAAAGCAGTCTTTTTCTCTTTCTCTTTTTCT
CTCTCCATTCTCTCCTCTCTTTTTTCATCAAAAAGGGGGGAGAGAAAAAGA
GAGAGGAGGGGGGG--
TCGAGGGAGAGAGGCTGTGACCAGGATTATTAAACAAAAAACCAAAACG
AGAATTCAACTTCGCGTTGGCCATTTTTTGCTTAAT-GGGGGGAGGTGG---
GTGTGGGTGGTGTGTGGCTCTCTCTCTGTGTGGT
ATATATATATGTATATTAGAAGTAGGTTGTGTGTGTGTGTATGTGTTTTAC
ACATATATATATCCGCGCCCTCACTCTCTCATATATAATTTATATGTACGC
ACAGAGAAAAAAGAGAGGCGCTCTCTTTTCCCCCCACCCCC-
GACAACCTTTGTTTACAGACCTGAGTGT

>U755

CTGGATCATTTTCCGATGATTACACCCAAAAAA---
ATATACAACTCGGGGAGACCTATGTATATATAT-----
GTAGGCCTTTCCCACATACACAGCAAAGTTTTGTACTCAAAATTTGCAGTA
AAAAAAAGGCCGAT
CGACGTTATAACGCACCGCCTATACAAAAGCAAAAATGTCCGTTTATACA
AAAAATATACGGCGTTTCGGTTTTTGGCGGGGTGGGTGCGTGTGTGGATA

ACGGCTCACATAACGTGTCGCGATGGATGACTTGGCTTCCTATTTCGTTGA
AGAACGCAGTAAAGTGCGATAAGTGGTATCAATTGCAGAATCATTCAATT
ACCGAATCTTTGAACGCAAACGGCGCATGGGAGAAGCTCTATTGTGTCAT
CCCCGTGCATGCCATATTCTCAGTGTCGAACAAAAAACAACACGCCGCCT
CCTCTCTTCTGCACATATATATAT---TATACCATACA
CAGTATATATATAATTATGTGTTGGAAGCCAAGAGGAGGCGTGTGTTTGT
GTTGTGCGCATATTATATGTATATATGCTGTGTGCACACGTAGACAAGTTA
GAGTTGGACAAATACACACATGCACTCTCTTTTGTGTGGGTGCGCGCGTG
GAAACTCCTCTCTGGTGCTTGCAAAGCAGTCTTTTTCTCTTTCTCTTTTTCT
CTCTCCATTCTCTCCTCTCTTTTTTCATCAAAAAGGGGGGAGAGAAAAAGA
GAGAGGAGGGGGGG--
TCGAGGGAGAGAGGCTGTGACCAGGATTATTAAACAAAAAACCAAAACG
AGAATTCAACTTCGCGTTGGCCATTTTTTGCTTAAT-GGGGGGAGGTGG---
GTGTGGGTGGTGTGTGGCTCTCTCTCTGTGTGGT
ATATATATATGTATATTAGAAGTAGGTTGTGTGTGTGTGTATGTGTTTTAC
ACATATATATATCCGCGCCCTCACTCTCTCATATATAATTTATATGTACGC
ACAGAGAAAAAAGAGAGGCGCTCTCTTTTCCCCCCACCCCC-
GACAACCTTTGTTTACAGACCTGAGTGT

>U883

CTGGATCATTTTCCGATGATTACACCCAAAAAA---
ATATACAACTCGGGGAGACCTATGTATATATAT-----
GTAGGCCTTTCCCACATACACAGCAAAGTTTTGTACTCAAAATTTGCAGTA
AAAAAAAGGCCGAT
CGACGTTATAACGCACCGCCTATACAAAAGCAAAAATGTCCGTTTATACA
AAAAATATACGGCGTTTCGGTTTTTGGCGGGGTGGGTGCGTGTGTGGATA
ACGGCTCACATAACGTGTCGCGATGGATGACTTGGCTTCCTATTTCGTTGA
AGAACGCAGTAAAGTGCGATAAGTGGTATCAATTGCAGAATCATTCAATT
ACCGAATCTTTGAACGCAAACGGCGCATGGGAGAAGCTCTATTGTGTCAT
CCCCGTGCATGCCATATTCTCAGTGTCGAACAAAAAACAACACGCCGCCT
CCTCTCTTCTGCACATATATATAT---TATACCATACA
CAGTATATATATAATTATGTGTTGGAAGCCAAGAGGAGGCGTGTGTTTGT
GTTGTGCGCATATTATATGTATATATGCTGTGTGCACACGTAGACAAGTTA
GAGTTGGACAAATACACACATGCACTCTCTTTTGTGTGGGTGCGCGCGTG
GAAACTCCTCTCTGGTGCTTGCAAAGCAGTCTTTTTCTCTTTCTCTTTTTCT
CTCTCCATTCTCTCCTCTCTTTTTTCATCAAAAAGGGGGGAGAGAAAAAGA
GAGAGGAGGGGGGG--
TCGAGGGAGAGAGGCTGTGACCAGGATTATTAAACAAAAAACCAAAACG
AGAATTCAACTTCGCGTTGGCCATTTTTTGCTTAAT-GGGGGGAGGTGG---
GTGTGGGTGGTGTGTGGCTCTCTCTCTGTGTGGT
ATATATATATGTATATTAGAAGTAGGTTGTGTGTGTGTGTATGTGTTTTAC
ACATATATATATCCGCGCCCTCACTCTCTCATATATAATTTATATGTACGC
ACAGAGAAAAAAGAGAGGCGCTCTCTTTTCCCCCCACCCCC-
GACAACCTTTGTTTACAGACCTGAGTGT

>U884

CTGGATCATTTTCCGATGATTACACCCAAAAAA---
ATATACAACTCGGGGAGACCTATGTATATATAT-----
GTAGGCCTTTCCCACATACACAGCAAAGTTTTGTACTCAAAATTTGCAGTA
AAAAAAAGGCCGAT
CGACGTTATAACGCACCGCCTATACAAAAGCAAAAATGTCCGTTTATACA
AAAAATATACGGCGTTTCGGTTTTTGGCGGGGTGGGTGCGTGTGTGGATA
ACGGCTCACATAACGTGTCGCGATGGATGACTTGGCTTCCTATTTCGTTGA
AGAACGCAGTAAAGTGCGATAAGTGGTATCAATTGCAGAATCATTCAATT
ACCGAATCTTTGAACGCAAACGGCGCATGGGAGAAGCTCTATTGTGTCAT
CCCCGTGCATGCCATATTCTCAGTGTCGAACAAAAAACAACACGCCGCCT
CCTCTCTTCTGCACATATATATAT---TATACCATACA
CAGTATATATATAATTATGTGTTGGAAGCCAAGAGGAGGCGTGTGTTTGT
GTTGTGCGCATATTATATGTATATATGCTGTGTGCACACGTAGACAAGTTA
GAGTTGGACAAATACACACATGCACTCTCTTTTGTGTGGGTGCGCGCGTG
GAAACTCCTCTCTGGTGCTTGCAAAGCAGTCTTTTTCTCTTTCTCTTTTTCT
CTCTCCATTCTCTCCTCTCTTTTTTCATCAAAAAGGGGGGAGAGAAAAAGA
GAGAGGAGGGGGGG--
TCGAGGGAGAGAGGCTGTGACCAGGATTATTAAACAAAAAACCAAAACG
AGAATTCAACTTCGCGTTGGCCATTTTTTGCTTAAT-GGGGGGAGGTGG---
GTGTGGGTGGTGTGTGGCTCTCTCTCTGTGTGGT
ATATATATATGTATATTAGAAGTAGGTTGTGTGTGTGTGTATGTGTTTTAC
ACATATATATATCCGCGCCCTCACTCTCTCATATATAATTTATATGTACGC
ACAGAGAAAAAAGAGAGGCGCTCTCTTTTCCCCCCACCCCC-
GACAACCTTTGTTTACAGACCTGAGTGT

>U975

CTGGATCATTTTCCGATGATTACACCCAAAAAA---
ATATACAACTCGGGGAGACCTATGTATATATAT-----
GTAGGCCTTTCCCACATACACAGCAAAGTTTTGTACTCAAAATTTGCAGTA
AAAAAAAGGCCGAT
CGACGTTATAACGCACCGCCTATACAAAAGCAAAAATGTCCGTTTATACA
AAAAATATACGGCGTTTCGGTTTTTGGCGGGGTGGGTGCGTGTGTGGATA
ACGGCTCACATAACGTGTCGCGATGGATGACTTGGCTTCCTATTTCGTTGA
AGAACGCAGTAAAGTGCGATAAGTGGTATCAATTGCAGAATCATTCAATT
ACCGAATCTTTGAACGCAAACGGCGCATGGGAGAAGCTCTATTGTGTCAT
CCCCGTGCATGCCATATTCTCAGTGTCGAACAAAAAACAACACGCCGCCT
CCTCTCTTCTGCACATATATATAT---TATACCATACA
CAGTATATATATAATTATGTGTTGGAAGCCAAGAGGAGGCGTGTGTTTGT
GTTGTGCGCATATTATATGTATATATGCTGTGTGCACACGTAGACAAGTTA
GAGTTGGACAAATACACACATGCACTCTCTTTTGTGTGGGTGCGCGCGTG
GAAACTCCTCTCTGGTGCTTGCAAAGCAGTCTTTTTCTCTTTCTCTTTTTCT
CTCTCCATTCTCTCCTCTCTTTTTTCATCAAAAAGGGGGGAGAGAAAAAGA
GAGAGGAGGGGGGG--
TCGAGGGAGAGAGGCTGTGACCAGGATTATTAAACAAAAAACCAAAACG
AGAATTCAACTTCGCGTTGGCCATTTTTTGCTTAAT-GGGGGGAGGTGG---
GTGTGGGTGGTGTGTGGCTCTCTCTCTGTGTGGT
ATATATATATGTATATTAGAAGTAGGTTGTGTGTGTGTGTATGTGTTTTAC
ACATATATATATCCGCGCCCTCACTCTCTCATATATAATTTATATGTACGC

ACAGAGAAAAAAGAGAGGCGCTCTCTTTTCCCCCCACCCCC-
GACAACCTTTGTTTACAGACCTGAGTGT

>U992

CTGGATCATTTTCCGATGATTACACCCAAAAAA---
ATATACAACTCGGGGAGACCTATGTATATATAT-----
GTAGGCCTTTCCCACATACACAGCAAAGTTTTGTACTCAAAATTTGCAGTA
AAAAAAAGGCCGAT
CGACGTTATAACGCACCGCCTATACAAAAGCAAAAATGTCCGTTTATACA
AAAAATATACGGCGTTTCGGTTTTGGCGGGGTGGGTGCGTGTGTGGATA
ACGGCTCACATAACGTGTCGCGATGGATGACTTGGCTTCCTATTTCGTTGA
AGAACGCAGTAAAGTGCGATAAGTGGTATCAATTGCAGAATCATTCAATT
ACCGAATCTTTGAACGCAAACGGCGCATGGGAGAAGCTCTATTGTGTCAT
CCCCGTGCATGCCATATTCTCAGTGTCGAACAAAAAACAACACGCCGCCT
CCTCTCTTCTGCACATATATATAT---TATACCATACA
CAGTATATATATAATTATGTGTTGGAAGCCAAGAGGAGGCGTGTGTTTGT
GTTGTGCGCATATTATATGTATATATGCTGTGTGCACACGTAGACAAGTTA
GAGTTGGACAAATACACACATGCACTCTCTTTTGTGTGGGTGCGCGCGTG
GAAACTCCTCTCTGGTGCTTGCAAAGCAGTCTTTTTCTCTTTCTCTTTTTCT
CTCTCCATTCTCTCCTCTCTTTTTTCATCAAAAAGGGGGGAGAGAAAAAGA
GAGAGGAGGGGGGG--
TCGAGGGAGAGAGGCTGTGACCAGGATTATTAAACAAAAAACCAAAACG
AGAATTCAACTTCGCGTTGGCCATTTTTTGCTTAAT-GGGGGGAGGTGG---
GTGTGGGTGGTGTGTGGCTCTCTCTCTGTGTGGT
ATATATATATGTATATTAGAAGTAGGTTGTGTGTGTGTGTATGTGTTTTAC
ACATATATATATCCGCGCCCTCACTCTCTCATATATAATTTATATGTACGC
ACAGAGAAAAAAGAGAGGCGCTCTCTTTTCCCCCCACCCCC-
GACAACCTTTGTTTACAGACCTGAGTGT

>ITS Type B (GenBank accession no. AJ000288)

CTGGATCATTTTCCGATGATTACACCCAAAAAA---
ATATACAACTCGGGGAGACCTATGTATATATAT-----
GTAGGCCTTTCCCACATACACAGCAAAGTTTTGTACTCAAAATTTGCAGTA
AAAAAAAGGCCGA
TCGACGTTATAACGCACCGCCTATACAAAAGCAAAAATGTCCGTTTATAC
AAAAAATATACGGCGTTTCGGTTTTGGCGGGGTGGGTGCGTGTGTGGAT
AACGGCTCACATAACGTGTCGCGATGGATGACTTGGCTTCCTATTTCGTTG
AAGAACGCAGTAAAGTGCGATAAGTGGTATCAATTGCAGAATCATTCAAT
TACCGAATCTTTGAACGCAAACGGCGCATGGGAGAAGCTCTATTGTGTCA
TCCCCGTGCATGCCATATTCTCAGTGTCGAACAAAAAACAACACGCCGCC
TCCTCTCTTCTGCACATATATATAT----TATACCATA
CACAGTATATATATAATTATGTGTTGGAAGCCAAGAGGAGGCGTGTGTTT
GTGTTGTGCGCATATTATATGTATATATGCTGTGTGCACACGTAGACAAGT
TAGAGTTGGACAAATACACACATGCACTCTCTTTTGTGTGGGTGCGCGCGT
GGAAACTCCTCTCTGGTGCTTGCAAAGCAGTCTTTTTCTCTTTCTCTTTTTC

TCTCTCCATTCTCTCCTCTCTTTTTTCATCAAAAAGGGGGGAGAGAAAAAG
AGAGAGGAGGGGGGGGGTCGAGGGAGAGAGGCTGTGACCAGGATTATTA
AACAAAAAACCAAAACGAGAATTCAACTTCGCGTTGGCCATTTTTTGCTT
AAT-GGGGGGAGGTGG---GTGTGGGTGGTGTGTGGCTCTCTCTCTGTGT
GGTATATATATATGTATATTAGAAGTAGGTTGTGTGTGTGTGTATGTGTTT
TACACATATATATATCCGCGCCCTCACTCTCTCATATATAATTTATATGTA
CGCACAGAGAAAAAAGAGAGGCGCTCTCTTTTCCCCCCACCCCC-
GACAACCNNTGTTTACAGACCTGAGTGT

>ITS Type C (GenBank accession no. AJ000294)

CTGGATCATTTTCCGATGATTACACCCAAAAAA---
ATATACAACTCGGGGAGACCTATGTATATATAT-----
GTAGGCCTTTCCCACACACACACAGCAAAGTTTTGTACTCAAAATTTGCAGTA
AAAAAA-GGCCGAT
CGACGTTATAACGCACCGCCTATACAAAAGCAAAAATGTCCGTTTATACA
AAAAATATACGGCGTTTCGGTTTTTGGCGGGGTGGGTGCGTGTGTGGATA
ACGGCTCACATAACGTGTCGCGATGGATGACTTGGCTTCCTATTTCGTTGA
AGAACGCAGTAAAGTGCGATAAGTGGTATCAATTGCAGAATCATTCAATT
ACCGAATCTTTGAACGCAAACGGCGCATGGGAGAAGCTCTATTGTGTCAT
CCCCGTGCATGCCATATTCTCAGTGTCGAACAAAAAACAACACGCCGCCT
CCTCTCTTCTGCACATATATATAT----TATACCATA
CACAGTATATATA—
ATTATGTGTTGGAAGCCAAGAGGAGGCGTGTGTTTGTGTTGTGCGCATATT
ATATGTATATATGCTGTGTGCACACGTAGACAAGTTAGAGTTGGACAAAT
ACACACATGCACTCTCTTTTGTGTGGGTGCGCGCGTGGAAACTCCTCTCTG
GTGCTTGCAAAGCAGTCTTTTTCTCTTTCTCTTTTTCTCTCTCCATTCTCTCC
TCTCTTTTTTCATCAAAAAGGGGGGAGAGAAAAAGAGAGAGGAGGGGG---
-
TCGAGGGAGAGAGGCTGTGACCAGGATTATTAAACAAAAAACCAAAACG
AGAATTCAACTTCGCGTTGGCCATTTTTTGCTTAAT-GGGGGGAGGTGG---
GTGTGGGTGGTGTGTGGCTCTCTCTCTGTGTGG
TATATATATATGTATATTAGAAGTAGGTTGTGTGTGTGTGTATGTGTTTTA
CACATATAT---CCGCGCC
CTCACTCTCTCATATATAATTTATATGTACGCACAGAGAAAAAAGAGAGG
CGCTCTCTTTTCCCCCCACCCCCCGACAACCTT-GTTTACAGACCTGAGTGT

>ITS Type LOMBARDI (GenBank accession no. AJ000295)

CTGGATCATTTTCCGATGATTACACCCAAAAAA---
ATATACAACTCGGGGAGACCTATGTATATATAT-----
GTAGGCCTTTCCCACATACACAGCAAAGTTTTGTACTCAAAATTTGCAGTA
AAAAAAAGGCCGAT
CGACGTTATAACGCACCGCCTATACAAAAGCAAAAATGTCCGTTTATACA
AAAAATATACGGCGTTTCGGTTTTTGGCGGGGTGGGTGCGTGTGTGGATA
ACGGCTCACATAACGTGTCGCGATGGATGACTTGGCTTCCTATTTCGTTGA
AGAACGCAGTAAAGTGCGATAAGTGGTATCAATTGCAGAATCATTCAATT

ACCGAATCTTTGAACGCAAACGGCGCATGGGAGAAGCTCTATTGTGTCAT
CCCCGTGCATGCCATATTCTCAGTGTCGAACAAAAAACAACACGCCGCCT
CCTCTCTTCTGCACATATATATATAT--TATACCATA
CACAGTATATATA--
ATTATGTGTTGGAAGCCAAGAGGAGGCGTGTGTTTGTGTTGTGCGCATATT
A
TATGTATATATGCTGTGTGCACACGTAGACAAGTTAGAGTTGGACAAATA
CACACATGCACTCTCTTTTGTGTGGGTGCGCGCGTGGAAACTCCTCTCTGG
TGCTTGCAAAGCAGTCTTTTTCTCTTTCTCTTTTTCTCTCTCCATTCTCTCCT
CTCTTTTTTCATCAAAAAGGGGGGAGAGAAAAAGAGAGAGGAGGGGG----
TCGAGGGAGAGAGGCTGTGACCAGGATTATTAAACAAAAAACCAAAACG
AGAATTCAACTTCGCGTTGGCCATTTTTTGCTTAAT-GGGGGGAGGTGG---
GTGTGGGTGGTGTGTGGCTCTCTCTCTGTGTGGT
ATATATATATGTATATTAGAAGTAGGTTGTGTGTGTGTGTATGTGTTTTAC
ACATATATATATCCGCGCCCTCACTCTCTCATATATAATTTATATGTACGC
ACAGAGAAAAAAGAGAGGCGCTCTCTTTTCCCCCCACCCCC-
GACAACCNNTGTTTACAGACCTGAGTGT

>ITS Type D (GenBank accession no. AJ634356)

CTGGATCATTTTCCGATGATTACACCCAAAAAA---
ATATACAACTCGGGGAGACCTATGTATATATAT-----
GTAGGCCTTTCCCACATACACAGCAAAGTTTTGTACTCAAAATTTGCAGTA
AAAAAA-GGCCGAT
CGACGTTATAACGCACCGCCTATACAAAAGCAAAAATGTCCGTTTATACA
AAAAATATACGGCGTTTCGGTTTTTGGCGGGGTGGGTGCGTGTGTGGATA
ACGGCTCACATAACGTGTCGCGATGGATGACTTGGCTTCCTATTTCGTTGA
AGAACGCAGTAAAGTGCGATAAGTGGTATCAATTGCAGAATCATTCAATT
ACCGAATCTTTGAACGCAAACGGCGCATGGGAGAAGCTCTATTGTGTCAT
CCCCGTGCATGCCATATTCTCAGTGTCGAACAAAAAACAACACGCCGCCT
CCTCTCTTCTGCACATATATATATAT----TATACCATA
CACAGTATATATA--
ATTATGTGTTGGAAGCCAAGAGGAGGCGTGTGTTTGTGTTGTGCGCATATT
AT
ATGTATATATGCTGTGTGCACACGTAGACAAGTTAGAGTTGGACAAATAC
ACACATGCACTCTCTTTTGTGTGGGTGCGCGCGTGGAAACTCCTCTCTGGT
GCTTGCAAAGCAGTCTTTTTCTCTTTCTCTTTTTCTCTCTCCATTCTCTCCTC
TCTTTTTTCATCAAAAAGGGGGGAGAGAAAAAGAGAGAGGAGGGGG----
TCGAGGGAGAGAGGCTGTGACCAGGATTATTAAACAAAAAACCAAAACG
AGAATTCAACTTCGC
GTTGGCCATTTTTTGCTTAAT-GGGGGGAGGTGG---
GTGTGGGTGGTGTGTGGCTCTCTCTCTGT
GTGGTATATATATATGTATATTAGAAGTAGGTTGTGTGTGTGT--
ATGTGTTTTACACATATAT----
CCGCGCCCTCACTCTCTCATATATAATTTATATGTACGCACAGAGAAAAA
AGAGAGGCGCTCTCTT

TTCCCCCCACCCCC-GACAACCTTTGTTTACAGACCTGAGTGT

>ITS Type E (GenBank accession no. AJ634367)

CTGGATCATTTTCCGATGATTACACC-AAAAAAAA-
CATATACAACTCGGGGAGACCTATGTATATA
TATATAT-
TAGGCCTTTCCCACATACACAGCAAAGTTTTGTACTCAAAATTTGCAGTAA
AAAAAAGGC
CGATCGACGTTATAACGCACCGCCTATACAAAAGCAAAAATGTCCGTTTA
TACAAAAAATATACGGCGTTTCGGTTTTTGGCGGGGTGGGTGCGTGTGTG
GATAACGGCTCACATAACGTGTCGCGATGGATGACTTGGCTTCCTATTTCG
TTGAAGAACGCAGTAAAGTGCGATAAGTGGTATCAATTGCAGAATCATTC
AATTACCGAATCTTTGAACGCAAACGGCGCATGGGAGAAGCTCTATTGTG
TCATCCCCGTGCATGCCATATTCTCAGTGTCGAACAAAAAACAACACGCC
GCCTCCTCTCTTCTGCACATATATATAT----TATAC
CATACACAGTATATATATAATTATGTGTTGGAAGCCAAGAGGAGGCGTGT
GTTTGTGTTGTGCGCATATTATATGTATATATGCTGTGTGCACACGTAGAC
AAGTTAGAGTTGGACAAATACACACATGCACTCTCTTTTGTGTGGGTGCG
CGCGTGGAAACTCCTCTCTGGTGCTTGCAAAGCAGTCTTTTTCTCTTTCTCT
TTTTCTCTCTCCATTCTCTCCTCTCTTTTTTCATCAAAAAGGGGGGAGAGA
AAAAGAGAGAGGAGGGGG----
TCGAGGGAGAGAGGCTGTGACCAGGATTATTAAACAAAAAACCAAAACG
AGAATTCAACTTCG
CGTTGGCCATTTTTTGCTTAATGGGGGGGAGGTGG---
GTGTGGGTGGTGTGTGGCTCTCTCTCTGTG
TGGTATATATATATGTATATTAGAAGTAGGTTGTGTGTGTGTGTATGTGTT
TTACACATATATATATCCGCGCCCTCACTCTCTCATATATAATTTATATGT
ACGCACAGAGAAAAAAGAGAGGCGCTCTCTTTTCCCCCCACCCCCCGACA
ACCTTTGTTTACAGACCTGAGTGT

>ITS Type F (GenBank accession no. AJ634373)

CTGGATCATTTTCCGATGATTACACC-
AAAAAAAAACATATACAACTCGGGGAGACCTATGTATATA
TATAT---
GTAGGCCTTTCCCACATACACAGCAAAGTTTTGTACTCAAAATTTGCAGTA
AAAAAA-GGC
CGATCGACGTTATAACGCACCGCCTATACAAAAGCAAAAATGTCCGTTTA
TACAAAAAATATACGGCGTTTCGGTTTTTGGCGGGGTGGGTGCGTGTGTG
GATAACGGCTCACATAACGTGTCGCGATGGATGACTTGGCTTCCTATTTCG
TTGAAGAACGCAGTAAAGTGCGATAAGTGGTATCAATTGCAGAATCATTC
AATTACCGAATCTTTGAACGCAAACGGCGCATGGGAGAAGCTCTATTGTG
TCATCCCCGTGCATGCCATATTCTCAGTGTCGAACAAAAAACAACACGCC
GCCTCCTCTCTTCTGCACATATATATAT----TATAC
CATACACAGTATATATATAATTATGTGTTGGAAGCCAAGAGGAGGCGTGT
GTTTGTGTTGTGCGCATATTATATGTATATATGCTGTGTGCACACGTAGAC
AAGTTAGAGTTGGACAAATACACACATGCACTCTCTTTTGTGTGGGTGCG

CGCGTGGAAACTCCTCTCTGGTGCTTGCAAAGCAGTCTTTTTCTCTTTCTCT
TTTTCTCTCTCCATTCTCTCCTCTCTTTTTTCATCAAAAAGGGGGGAGAGA
AAAAGAGAGAGGAGGGGG----
TCGAGGGAGAGAGGCTGTGACCAGGATTATTAAACAAAAAACCAAAACG
AGAATTCAACTTC
GCGTTGGCCATTTTTTGCTTAATGGGGGGGAGGTGG---
GTGTGGGTGGTGTGTGGCTCTCTCTCTG
TGTGGTATATATATATGTATATTAGAAGTAGGTTGTGTGTGTGTGTATGTG
TTTTACACATATATATATCCGCGCCCTCACTCTCTCATATATAATTTATATG
TACGCACAGAGAAAAAAGAGAGGCGCTCTCTTTTCCCCCCACCCCCCGAC
AACCTTTGTTTACAGACCTGAGTGT

>ITS Type G (GenBank accession no. AJ000297)

CTGGATCATTTTCCGATGATTACACC-AAAAAAA--
CATATACAACTCGGGGAGACCTATGTATATA
TATAT---
GTAGGCCTTTCCCACATACACAGCAAAGTTTTGTACTCAAAATTTGCAGTA
AAAAAA-GGC
CGATCGACGTTATAACGCACCGCCTATACAAAAGCAAAAATGTCCGTTTA
TACAAAAAATATACGGCGTTTCGGTTTTTGGCGGGGTGGGTGCGTGTGTG
GATAACGGCTCACATAACGTGTCGCGATGGATGACTTGGCTTCCTATTTCG
TTGAAGAACGCAGTAAAGTGCGATAAGTGGTATCAATTGCAGAATCATTC
AATTACCGAATCTTTGAACGCAAACGGCGCATGGGAGAAGCTCTATTGTG
TCATCCCCGTGCATGCCATATTCTCAGTGTCGAACAAAAAACAACACGCC
GCCTCCTCTCTTCTGCACATATATATAT----TATAC
CATACACAGTATATATATATAATTATGTGTTGGAAGCCAAGAGGAGGCGTGT
GTTTGTGTTGTGCGCATATTATATGTATATATGCTGTGTGCACACGTAGAC
AAGTTAGAGTTGGACAAATACACACATGCACTCTCTTTTGTGTGGGTGCG
CGCGTGGAAACTCCTCTCTGGTGCTTGCAAAGCAGTCTTTTTCTCTTTCTCT
TTTTCTCTCTCCATTCTCTCCTCTCTTTTTTCATCAAAAAGGGGGGAGAGA
AAAAGAGAGAGGAGGTGG----
TCGAGGGAGAGAGGCTGTGACCCGGATTATTAAACAAAAAACCAAAACG
AGAATTCAACTTC
GCGTTGGCCATTTTTTGCTTAAT-
GGGGGGAGGTGGTGGGTGTGGGTGGTGTGTGGCTCTCTCTCTG
TGTGGTATATATATATGTATATTAGAAGTAGGTTGTGTGTGTGTGTATGTG
TTTTACACATATATATATCCGCGCCCTCACTCTCTCATATATAATTTATATG
TACGCACAGAGAAAAAAGAGAGGCGCTCTCTTTTCCCCCCACCCCCCGAC
AACCTT-GTTTACAGACCTGAGTGT

>ITS Type H (GenBank accession no. AJ634376)

CTGGATCATTTTCCGATGATTACACC-AAAAAAA--
CATATACAACTCGGGGAGACCTATGTATATATA
TAT---
GTAGGCCTTTCCCACATACACAGCAAAGTTTTGTACTCAAAATTTGCAGTA
AAAAAA-GGCCG

ATCGACGTTATAACGCACCGCCTATACAAAAGCAAAAATGTCCGTTTATA
CAAAAAATATACGGCGTTTCGGTTTTTGGCGGGGTGGGTGCGTGTGTGGA
TAACGGCTCACATAACGTGTCGCGATGGATGACTTGGCTTCCTATTTCGTT
GAAGAACGCAGTAAAGTGCGATAAGTGGTATCAATTGCAGAATCATTCAA
TTACCGAATCTTTGAACGCAAACGGCGCATGGGAGAAGCTCTATTGTGTC
ATCCCCGTGCATGCCATATTCTCAGTGTCGAACAAAAAACAACACGCCGC
CTCCTCTCTTCTGCACATATATATATATATTATACCATACACAGTATATAT
ATAATTATGTGTTGGAAGCCAAGAGGAGGCGTGTGTTTGTGTTGTGCGCA
TATTATATGTATATATGCTGTGTGCACACGTAGACAAGTTAGAGTTGGACA
AATACACACATGCACTCTCTTTTGTGTGGGTGCGCGCGTGGAAACTCCTCT
CTGGTGCTTGCAAAGCAGTCTTTTTCTCTTTCTCTTTTTCTCTCTCCATTCTC
TCCTCTCTTTTTTCATCAAAAAGGGGGGAGAGAAAAAGAGAGAGGAGGTG
G----
TCGAGGGAGAGAGGCTGTGACCAGGATTATTAAACAAAAAACCAAAACG
AGAATTCAACTTCGC
GTTGGCCATTTTTTGCTTAAT-GGGGGGAGGTGG---
GTGTGGGTGGTGTGTGGCTCTCTCTCTGTGT
GGTATATATATATGTATATTAGAAGTAGGTTGTGTGTGTGTGTATGTGTTT
TACACATATATATATCCGCGCCCTCACTCTCTCATATATAATTTATATGTA
CGCACAGAGAAAAAAGAGAGGCGCTCTCTTTTCCCCCCACCCCCCGACAA
CCTTTGTTTACAGACCTGAGTGT
